# Hereditary neuropathy with liability to pressure palsies (HNPP): Intrafamilial phenotypic variability and early childhood refusal to walk as the presenting symptom

**DOI:** 10.1186/s13052-022-01280-z

**Published:** 2022-06-03

**Authors:** Shani Karklinsky, Shir Kugler, Omer Bar-Yosef, Andreea Nissenkorn, Anat Grossman-Jonish, Irit Tirosh, Asaf Vivante, Ben Pode-Shakked

**Affiliations:** 1 Department of Pediatrics B, Edmond and Lily Safra Children’s Hospital, Sheba Medical Center, 52621 Tel-Hashomer, Israel; 2 Sackler Faculty of Medicine, Tel-Aviv University, Tel-Aviv, Israel; 3 Pediatric Neurology Unit, Edmond and Lily Safra Children’s Hospital, Sheba Medical Center, Tel-Hashomer, Israel; 4 Talpiot Medical Leadership Program, Sheba Medical Center, Tel-Hashomer, Israel; 5 Center for Rare Disorders-Magen, Wolfson Medical Center, Holon, Israel; 6 The Danek Gertner Institute of Human Genetics, Sheba Medical Center, Tel-Hashomer, Israel; 7 Pediatric Rheumatology Unit, Edmond and Lily Safra Children’s Hospital, Sheba Medical Center, Tel-Hashomer, Israel; 8 Pediatric Nephrology Unit, Edmond and Lily Safra Children’s Hospital, Sheba Medical Center, Tel-Hashomer, Israel

**Keywords:** Hereditary neuropathy with liability to pressure palsies (HNPP), PMP22, The limping child

## Abstract

**Background:**

Limping and/or refusal to walk is a common complaint in the setting of the pediatric department, with a widely diverse differential diagnosis. An unusual etiology, is that of a hereditary neuropathy.

Hereditary neuropathy with liability to pressure palsies (HNPP) is a recurrent, episodic demyelinating neuropathy, most commonly caused by a 17p11.2 chromosomal deletion encompassing the *PMP22* gene.

**Methods:**

We pursued chromosomal microarray analysis (CMA) in multiple affected individuals of a single extended family, manifesting a range of phenotypic features consistent with HNPP.

**Results:**

A 4.5 years-old boy presented for in-patient evaluation due to refusal to walk. Initial investigations including spine MRI and bone scan failed to yield a conclusive diagnosis. Following family history, which implied an autosomal dominant mode of inheritance, CMA was pursued and confirmed a 17p11.2 deletion in the proband consistent with HNPP. Importantly, following this diagnosis, four additional affected family members were demonstrated to harbor the deletion. Their variable phenotypic features, ranging from a prenatal diagnosis of a 6 months-old sibling, to recurrent paresthesias manifesting in the fourth decade of life, are discussed.

**Conclusions:**

Our experience with the family reported herein demonstrates how a thorough anamnesis can lead to a rare genetic etiology with a favorable prognosis and prevent unnecessary investigations, and underscores HNPP as an uncommon diagnostic possibility in the limping child.

## Background

Limping and/or refusal to walk is a common complaint in the setting of the pediatric department. The differential diagnosis is diverse, and includes infectious, inflammatory, traumatic and neoplastic etiologies, among others. An unusual etiology, especially in the pediatric population, is that of a hereditary neuropathy.

Hereditary neuropathy with liability to pressure palsies (HNPP) is a recurrent, episodic demyelinating neuropathy, characterized by acute onset of a non-painful focal sensory and motor neuropathy in a single nerve. The peroneal and ulnar nerves are most frequently affected nerves [1]. Compression or mild pressure over years to decades and trauma are the main triggers for the symptoms [2]. HNPP may have an indolent course over years to decades.

HNPP is an autosomal-dominant disorder, with 80% of patients harboring a 17p11.2 chromosomal deletion, encompassing the peripheral myelin protein 22 (*PMP22*) gene. The PMP22 protein is produced by Schwann cells which are responsible for myelin production. While its exact role is yet to be elucidated, evidence suggests that PMP22 plays an important role in myelin composition and activity [3]. Pathogenic variants or copy number variants in *PMP22* are responsible for the most common hereditary neuropathy - Charcot Marie Tooth type 1A. The latter is known to be caused by a duplication of 17p11.2 encompassing *PMP22* [4].

The HNPP phenotype usually first manifests in the 2nd or 3rd decade of life, however the age range is variable beginning at infancy and reaching well into adulthood [1]. The phenotypic presentation is diverse as well, and many individuals remain asymptomatic.

This diagnostic possibility should be suspected in any patient with recurrent focal compression neuropathies and a family history consistent with autosomal dominant inheritance. Other supporting findings can be physical examination findings showing previous nerve palsy (i.e. focal weakness, atrophy or sensory loss), diffusely reduced tendon reflexes and mild to moderate pes cavus foot deformity [2]. The definitive diagnosis is achieved by genetic analysis. Treatment is mostly supportive and focuses on reduction of risk factors and triggers, such as prolonged cross-legged sitting, prolonged leaning on elbows or rapid weight loss.

Overall, HNPP has a favorable prognosis. Full recovery is expected over a period of days to months, while in a small percentage of cases there might be incomplete resolution, however, persistent symptoms are rarely severe.

We report herein our experience with an unusual presentation of HNPP as refusal to walk in early childhood, and discuss the insights gained from this rare clinical scenario, leading to the identification of at least five affected individuals in the extended family, demonstrating extremely variable phenotypes.

## Methods

### Patient recruitment

Patients III:4, II:3 and III:1 were evaluated and underwent genetic counselling at the Pediatric Genetics clinic, the Institute for Rare Diseases, Edmond and Lily Safra Children’s Hospital, Sheba Medical Center, as part of their clinical evaluation.

### Chromosomal microarray analysis (CMA)

Patient blood samples were analyzed using array comparative genomic hybridization (CGH) V8.1 and V7.6 custom-designed oligonucleotide arrays, BCM (Baylor College of Medicine), manufactured by Agilent Technologies, Inc. (Santa Clara, CA). CMA was performed according to the manufacturers’ instructions. The array CGH procedure, data analysis, and reporting have been previously described [5, 6].

## Results

### Clinical presentations

#### Patient III:4

The proband, a previously healthy 4 years-old boy presented to the Pediatric Department following a 2 weeks’ history of bilateral non-specific leg pain. A week prior he had a few days of high fevers without additional signs or symptoms. Three days prior to his admission he started complaining of nonspecific leg pain that deteriorated up to the point that he refused to walk. Due to elevated serum C-reactive protein (CRP) obtained during initial investigations done by his primary pediatrician he was referred to our hospital for further workup.

Upon his admission, his physical exam was considered normal with no signs of arthritis, edema, or limitation in range of motion of his limbs. In addition, thorough neurological exam was unremarkable despite his refusal to walk. Subsequent laboratory studies showed normal complete blood count (CBC) and chemistry, elevated CRP, normal blood smear, negative blood culture results as well as normal serum immunoglobulins and complement levels. The patient completed left leg radiographs which were normal, and a bone scan which demonstrated a non-specific decreased absorption in his left foot. Consequently, he was treated with non-steroidal anti-inflammatory drugs (NSAIDs) which resulted in clinical improvement as he started walking and serum CRP normalized. The patient was discharged home.

One week following his discharge, the patient returned to the orthopedic clinic for a follow up examination, and presented with left foot weakness, absent achilles reflex and sensory impairment. He was re-admitted to the Pediatric Department. His lab results were normal, and a lumbrosacral magnetic resonance imaging (MRI) was considered normal as well.

**Fig. 1:**
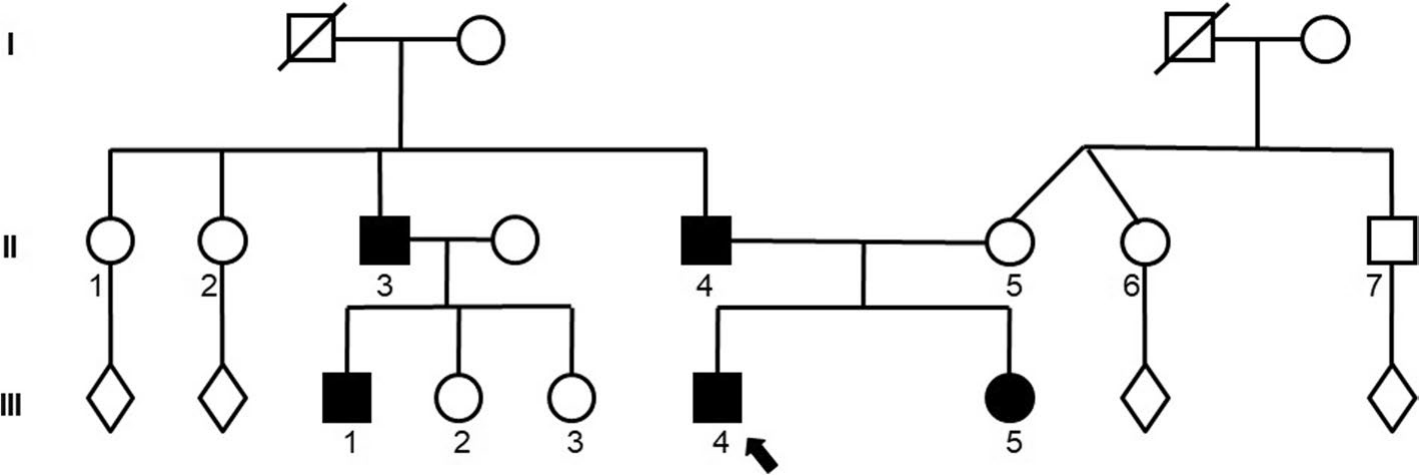
Family pedigree of multiple-affected family with Hereditary Neuropathy with Liability to Pressure Palsies (HNPP). Full symbols designate affected individuals. Arrow designates the proband

Thorough family history taking revealed that both the patient’s father and his parental uncle reported recurrent episodes of paresthesia and loss of sensation over their palms (Fig. 1). Further family investigation revealed that the patient’s reportedly healthy 6 months-old sister (patient III:5), who underwent prenatal chromosomal microarray analysis (CMA) testing harbored a genetic deletion in 17p11.2, encompassing the *PMP22* gene, which is associated with Hereditary neuropathy with liability to pressure palsies syndrome (HNPP). This finding brought to the working diagnosis of HNPP in the proband, as well as his father, uncle and male cousin.

#### Patient II:3

The 51 years-old paternal uncle of the proband, was reportedly asymptomatic until the age of ~ 30 years, when he began experiencing episodes of numbness, loss of sensation and control of this arm and hand, which would wake him up from sleep. Initially, these symptoms, which would resolve within 10–15 minutes, are attributed to a motor vehicle accident he had several years prior. While these episodes ultimately resolved, he complained of numbness of both legs after prolonged sitting (e.g. on the toilet), often with subsequent limping or foot dragging. Similar symptoms would be triggered by cross-legged sitting. He had previously undergone extensive orthopedic and neurological investigations which failed to yield a diagnosis.

#### Patient II:4

The 41 years-old father of the proband reported recurring episodes of numbness and loss of sensation in his fingers, triggered by pressure, and was otherwise reportedly healthy.

#### Patient III:1

The 18 years-old male paternal cousin of the proband, was reportedly asymptomatic until the age of 17.5 years, when he began experiencing repeated episodes of burning sensation in both feet (left more than right), triggered by prolonged standing. The painful paraesthesias precluded him from enjoying his hobby as a basketball player. Neurological investigations were underway, and had included electromyography (EMG) which was reported to be consistent with bilateral sensorimotor polyneuropathy in both feet.

### Chromosomal microarray analysis (CMA)

CMA was pursued for all symptomatic family members (Proband III:7, as well as II:3, II:4, III:3) and confirmed the 17p11.2 deletion [arr: 17p12(14,100,11-15,422,557) × 1] to completely segregate with the phenotype in the family. Of note, the paternal grandfather of the proband (and father of II:3 and II:4) had died of a liver malignancy at 50 years of age, and was therefore unavailable for CMA.

Following the cytogenetic diagnosis of HNPP for the affected individuals in the family, further unnecessary tests were avoided, not only for the proband but also for his father, uncle and cousin. In addition, thorough genetic counselling was enabled. The proband experienced complete resolution of his symptoms several months after discharge.

## Discussion

The differential diagnosis of the limping child is extremely wide and diverse, and spans a multitude of etiologies and systems, including infectious, inflammatory, traumatic, and neoplastic – as well as other, less common – causes [7]. Of these, mono- and polyneuropathies comprise a subgroup of both hereditary and acquired disorders, which often overlap and may initially be clinically indistinguishable in terms of signs and symptoms. Acquired disorders might include compression neuropathies, chronic inflammatory demyelinating polyneuropathy (CIDP), and Guillain-Barré Syndrome [8], among others. Hereditary causes include Charcot-Marie-Tooth and other hereditary polyneuropathies [9], however these might often be overlooked as a diagnostic possibility in the common pediatric presentation of the limping child.

The prevalence of hereditary neuropathy with liability to pressure palsies (HNPP) is estimated at 7–16:100,000 individuals [10], making it a rare entity. Nevertheless, it is widely familiar to pediatric and adult neurologists as part of the differential diagnosis of compressive neuropathies. The family reported herein, and especially the phenotypic manifestations of the proband, highlight several insights for clinicians who may encounter such patients.

A feature of HNPP demonstrated by this multiplex family is the intrafamilial phenotypic variability, which is consistent with previous reports [11–13]. This includes variability both in age of symptom onset and in the nature of phenotypic expression, ranging from a prenatal diagnosis in an asymptomatic 6 months old sibling, to early-childhood onset in the 4 years-old proband, and to diverse compression-triggered neuropathic complaints in the fourth decade of life in his father and uncle. In addition, the myriad of possible clinical manifestations, including mono- and polyneuropathies, upper and lower extremity involvement, are underscored. The combination of each of these with the family history consistent with an autosomal dominant mode of inheritance, served as the key clue leading to the diagnosis.

While the first onset of symptoms in HNPP is generally in the second or third decade of life, it can occur at any age, and with the growing availability of molecular studies, early onset cases are more commonly reported. In the largest childhood-onset series of cases to date (*n* = 12), Chrestian et al. noted that peroneal nerve palsy was the most common presentation (42%), followed by brachial plexus palsy (24%) [14]. They suggested that any unexplained mononeuropathy or multifocal neuropathy in children should raise clinical suspicion of HNPP. In this context, the fact that the proband reported herein presented before 5 years of age is not unusual, and further illustrates their point that early and timely diagnosis can facilitate not only appropriate genetic counselling but also affect the clinical management and prevent unwarranted tests.

Further, diagnosis of a genetic etiology in the in-patient evaluation of a child with a common compliant, brought in the family reported herein to several clinical implications, well beyond the course of evaluation and management of the proband. First, it allowed the medical team to reassess and forgo unnecessary tests and procedures. Second, it enabled a timely diagnosis of multiple affected individuals in the family, spanning at least two generations and a wide range of ages, and affected the course of their medical investigations as well, which in some cases were prolonged and ongoing diagnostic odysseys involving multidisciplinary specialties. Finally, parents, siblings and additional at-risk individuals in the family were able to receive accurate genetic counselling.

## Conclusions

Our experience with the multiplex family reported herein, underscores how a common presentation in pediatrics (‘the limping child’), may lead to a genetic etiology, and how a cytogenetic diagnosis in the pediatric inpatient setting may lead to the identification of numerous additional affected family members over several generations.

## Data Availability

The datasets used during the current study are available from the corresponding author upon reasonable request.

## Abbreviations

CBC: Complete blood count
CGH: Comparative genomic hybridization
CMA: Chromosomal microarray analysis
CRP: C-reactive protein
EMG: Electromyography
HNPP: Hereditary neuropathy with liability to pressure palsies
MRI: Magnetic resonance imaging
NSAIDs: Non-steroidal anti-inflammatory drugs
PMP22: Peripheral myelin protein 22
